# Ethanol Infusion Into the Vein of Marshall for Atrial Fibrillation: Clinical Efficacy and Technical Limitations

**DOI:** 10.1002/clc.70398

**Published:** 2026-06-29

**Authors:** Xuepeng Zheng, Hongliang Yang, Huan Sun, Ming Yu, Lujia Ni, Daoyuan Si, Yuquan He

**Affiliations:** ^1^ Department of Cardiology, China‐Japan Union Hospital of Jilin University Jilin Provincial Molecular Biology Research Center for Precision Medicine of Major Cardiovascular Disease Changchun China; ^2^ Department of Ultrasonography China‐Japan Union Hospital of Jilin University Changchun China

**Keywords:** atrial fibrillation, catheter ablation, ethanol, mitral isthmus, vein of Marshall

## Abstract

**Background:**

Despite advancements in radiofrequency ablation (RFCA) technology and strategy, the long‐term rhythm outcomes of persistent atrial fibrillation (PeAF) remain suboptimal. Since the vein of Marshall (VOM) is situated within the mitral isthmus (MI) area and covers local neural innervation and fiber networks, its pathophysiological role in mediating and maintaining atrial fibrillation (AF) and causing arrhythmia recurrence has garnered increasing attention.

**Hypothesis:**

Ethanol infusion into the vein of Marshall (EIVOM) may complement RFCA by delivering anatomically targeted chemical injury along the VOM course, thereby facilitating contiguous MI lesion formation and durable MI block.

**Methods:**

We performed a narrative review of mechanistic, procedural, and clinical evidence regarding EIVOM in AF ablation, with emphasis on MI block, rhythm outcomes, technical limitations, complications, and its potential role in pulsed field ablation (PFA)‐based workflows.

**Results:**

Available evidence indicates that EIVOM facilitates durable MI block by targeting VOM‐related epicardial connections and may improve rhythm outcomes when combined with RFCA in selected PeAF ablation strategies. Its clinical application is influenced by procedural and anatomical factors, while safety considerations and its potential complementary role in PFA‐based workflows remain important areas for further evaluation.

**Conclusions:**

EIVOM is a promising adjunct to RFCA, particularly for facilitating durable MI block and reducing residual epicardial conduction. Further standardized multicenter studies are needed to refine patient selection, procedural integration, safety optimization, and its role in contemporary AF ablation.

## Graphical abstract

Evaluating EIVOM in atrial fibrillation: Clinical efficacy, mechanistic insights, and technical considerations. Abbreviations: AF, atrial fibrillation; AT, atrial tachycardia; CS, coronary sinus; EIVOM, ethanol infusion into the vein of Marshall; LAA, left atrial appendage; LIPV, left inferior pulmonary vein; MA, mitral annulus; MI, mitral isthmus; RF, radiofrequency energy; VOM, vein of Marshall.

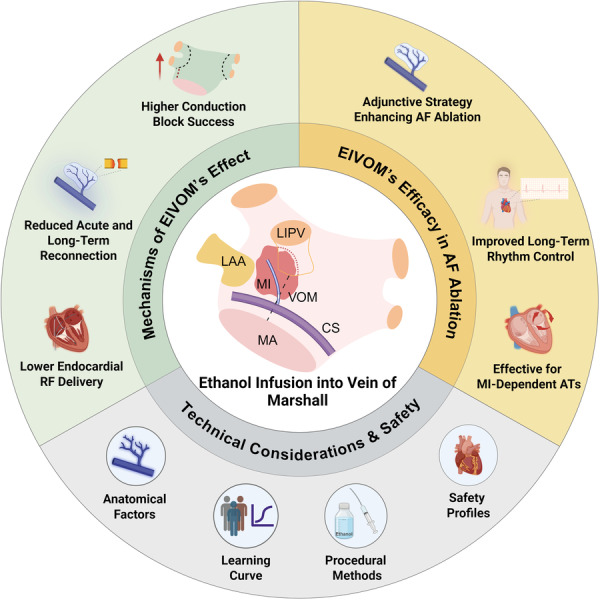

**Abbreviations**
AF: atrial fibrillation
AT: atrial tachycardia
CS: coronary sinus
EIVOM: ethanol infusion into the vein of Marshall
GCV: great cardiac vein
ICE: intracardiac echocardiography
LA: left atrial
LAA: left atrial appendage
LIPV: left inferior pulmonary vein
LPV: left pulmonary vein
MA: mitral annulus
MB: Marshall bundle
MI: mitral isthmus
MIBB: mitral isthmus bidirectional block
PeAF: persistent atrial fibrillation
PFA: pulsed field ablation
PMF: perimitral flutter
PVI: pulmonary vein isolation
RF: radiofrequency energy
RFCA: radiofrequency ablation
VOM: vein of Marshall

## 1 Introduction

While mitral isthmus (MI) bidirectional conduction block has been demonstrated to be valuable in atrial compartmentalization strategy and recurrence control for persistent atrial fibrillation (PeAF), achieving durable block remains challenging [1, 2]. The MI region combines thick myocardium, epicardial adipose, and convective cooling from the coronary sinus (CS) and adjacent vessels, which favor incomplete endocardial radiofrequency lesions [3, 4]. The vein of Marshall (VOM), an embryological remnant typically coursing from the CS to the left atrial (LA) ridge, harbors abundant autonomic innervation and muscular connections that can trigger and maintain AF and provide epicardial connections across the MI [5, 6, 7]. EIVOM produces transmural chemical injury along the MI, creating a targeted lesion despite local anatomic complexity and eliminating epicardial conduction gaps simultaneously, thereby facilitating durable MI block [8].

Contemporary investigations suggest that integrating ethanol infusion into the vein of Marshall (EIVOM) with radiofrequency catheter ablation (RFCA) improves rhythm outcomes over RFCA alone. However, substantial ambiguity persists regarding EIVOM's therapeutic benefits in optimizing clinical outcomes for PeAF, its contribution to achieving durable MI block, its procedure‐specific constraints and safety profile, and its future role in the emerging era of pulsed field ablation (PFA). Building on this, this review aims to synthesize the mechanistic basis of the technique, critically appraise its efficacy in atrial fibrillation (AF) management, and delineate technical limitations, safety considerations, and evolving role across contemporary ablation strategies.

## 2 Mechanisms of EIVOM's Therapeutic Role in AF

### 2.1 Impact of EIVOM on MI Block

**Figure 1:**
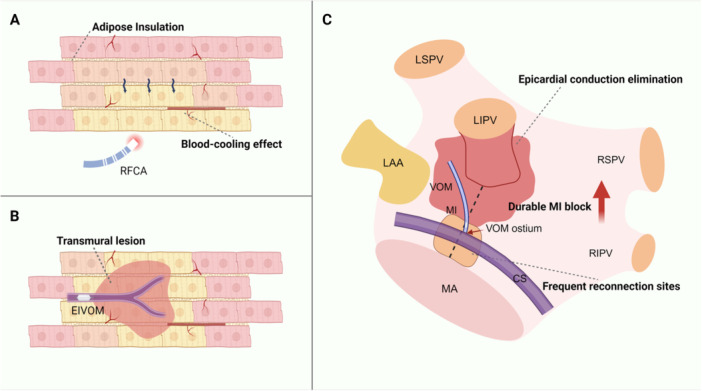
Anatomical basis and transmural lesion patterns produced by EIVOM. (A) RFCA lesion formation is limited by thick atrial myocardium, surrounding epicardial fat, and the cooling effect of the coronary sinus and adjacent arterial blood flow. (B) Ethanol infusion into the VOM produces consistent transmural myocardial injury along the VOM course. (C) Resulting lesions predominantly involve the LIPV and the LIPV‐adjacent segment of the MI, facilitating effective ablation of the VOM region and promoting durable MI block. The areas between the MA and the VOM ostium are highlighted, as these regions represent common sites of residual conduction after EIVOM and often need targeted complementary ablation. CS, coronary sinus; EIVOM, ethanol infusion into the vein of Marshall; LAA, left atrial appendage; LIPV, left inferior pulmonary vein; LSPV, left superior pulmonary vein; MA, mitral annulus; MI, mitral isthmus; RF, radiofrequency; RFCA, radiofrequency catheter ablation; RIPV, right inferior pulmonary vein; RSPV, right superior pulmonary vein; VOM, vein of Marshall.

The MI represents a challenging target for a durable bidirectional block due to local anatomic complexity and VOM‐related epicardial conduction [9, 10]. The Marshall bundle (MB), an epicardial myocardial structure closely associated with the VOM, may form multiple electrical connections with the LA myocardium and CS musculature, providing epicardial pathways that bypass an apparently continuous endocardial MI lesion [11]. Owing to its epicardial location, adipose insulation, and distance from the endocardium, endocardial RFCA may fail to create fully transmural lesions or may eliminate only the endocardial breakout while leaving the protected epicardial component intact [12]. In contrast, EIVOM delivers ethanol through the VOM lumen and produces anatomically targeted chemical injury from the venous‐epicardial side along the VOM/MB course, helping create a more contiguous MI lesion set and eliminate MB‐related epicardial connections and VOM‐related ectopic activity, thereby facilitating durable MI bidirectional block (MIBB) in AF ablation (Figure 1). This advantage reflects the anatomy‐dependent lesion distribution of EIVOM. Despite the heterogeneity across studies, the extensive damage predominantly involves the left pulmonary vein (LPV) side of the MI lesion. Recurrence analyses indirectly corroborate this pattern—MI reconnection sites were more often observed in the mitral annulus (MA) aspect, both endocardially and epicardially [13, 14, 15]. Likewise, Zuo et al. showed that acute MI recovery localizes to the MI region after RFCA alone, but shifts to the MA aspect and epicardial CS after EIVOM [16]. Furthermore, residual MI conduction after EIVOM may involve the CS and the great cardiac vein (GCV) musculature. Schurmann et al. showed that recurrent MI conduction after EIVOM was frequently related to MA‐side and CS‐associated gaps [17]. Pambrun et al. demonstrated that residual GCV‐related epicardial gaps after VOM ethanol infusion and endocardial MI ablation were observed in nearly half of the patients and were often eliminated by endovascular ablation within the first centimeter of the GCV [18]. Clinically, these findings indicate that detailed electrophysiological assessment should be performed after EIVOM‐assisted MI ablation, with particular attention to annular‐side gaps, the VOM ostium region, and CS‐GCV myocardial sleeves, while remaining alert to alternative pathways suggested by activation patterns. Failed MI block after technically successful EIVOM should therefore prompt systematic mapping of residual conduction rather than empirical ablation alone. Targeted MA‐side reinforcement and, when necessary, cautious intra‐CS/GCV ablation may help achieve complete MIBB. Beyond the distribution of residual gaps, lesion durability is another pivotal factor contributing to the efficacy of EIVOM. While the duration of radiofrequency lesions varies depending on ablation parameters (such as power, duration, and contact force) and atrial tissue characteristics, EIVOM achieves efficient and transmural ethanol penetration, promoting more sustainable lesion formation. Laredo et al. performed LA bipolar voltage mapping before and after EIVOM in 256 patients undergoing de novo ablation and in 24 patients undergoing redo procedures, showing that EIVOM‐related lesions remained intact in all cases with recurrence, thereby supporting its role in minimizing reconduction [19].

Accordingly, the adequate ablation by EIVOM significantly enhances MI block rates and reduces both acute and long‐term recurrence (Table 1). Zuo et al. reported superior outcomes after EIVOM, showing significantly lower acute MI reconduction in the EIVOM versus RFCA group (6.7% vs. 35.1%) and higher final MIBB improvement (97.8% vs. 72.7%, *p* < 0.01) [16]. Across prospective cohorts, EIVOM consistently increased the MI block rate by approximately 20% compared with RFCA [20, 25]. Moreover, EIVOM yielded a more durable MI block, with lower reconnection than RFCA alone (14.3% vs. 37.0%; *p* = 0.026) over 291 ± 170 days of follow‐up [24]. Beyond improving MIBB and reducing recurrence, EIVOM's robust transmural lesions lead to the lower endocardial RFCA burden, enabling MI block with fewer and shorter radiofrequency applications [26, 27]. It is also associated with lower mean contact force (*p* < 0.01) and fewer high–contact‐force applications compared with RFCA alone (7.9 ± 1.8 vs. 13.7 ± 3.5; *p* = 0.03) [28].

**Table 1:** Impact of EIVOM on MI block achievement.

Author	Year	Patient	MI procedural parameters (E vs. R)				
			Successful block rate	RFCA application time	Reconnection rate		
					Acute^a^	Long‐term and follow‐up^b^	
Zuo S [16]	2024	PeAF	97.7% (44/45) versus 72.1% (32/44), *p* < 0.01	348.0 ± 58.1 versus 142.2 ± 46.6 s, *p* < 0.01	6.7% (3/45) versus 35.1% (13/44), *p* < 0.01	—	—
Ge WL [20]	2024	PeAF	96% (69/72) versus 76% (35/46), *p* < 0.01	—	—	—	—
Huang L [21]	2023	PeAF	95.6% (129/135) versus 87.6% (156/178), *p* = 0.015	6.6 ± 4.4 versus 7.8 ± 4.3 min, *p* = 0.013	12.6% (17/135) versus 30.9% (55/178), *p* < 0.001	—	—
Lai Y [22]	2021	PeAF	95.5% (63/66) versus 80.8% (101/125), *p* < 0.01	7.0 ± 3.5 versus 11.8 ± 3.5 min, *p* < 0.001	—	—	—
Ishimura M [23]	2021	PeAF(First and redo ablation)	97% (171/176) versus 92% (353/384), *p* = 0.09 in first; 0.10 in redo	—	—	58% (25/43) versus 49% (39/80), *p* = 0.32	E: 335 ± 239 days; R: 418 ± 336 days
Nakashima T [24]	2020	PaAF PeAF	98.7% (150/152) versus 63.6% (70/110), *p* < 0.001	5.0 (3.0–7.0) versus 19.0 (13.6–22.0) min, *p* < 0.001	—	37.1% (3/35) versus 67.4% (31/46), *p* = 0.008	291 ± 170 days

*Note:* Data are presented as *n*%, *n*/*N*, mean ± SD, and M (IQR).

Abbreviations: E, EIVOM group plus RFCA; EIVOM, ethanol infusion into the vein of Marshall; m, month; MI, mitral isthmus; min, minute; PaAF, paroxysmal atrial fibrillation; PeAF, persistent atrial fibrillation; PMF, peri‐mitral flutter; R, only RFCA group; RFCA, radiofrequency catheter ablation; s, second.

^a^ Acute reconnection was measured after 30 min of MI bidirectional block.

^b^ Long‐term MI reconnection was measured among patients who underwent a redo procedure due to recurrence.

However, contrasting findings have been reported. In a cohort by Ishimura et al., adjunctive EIVOM did not yield statistically significant improvements in the MIBB rate or long‐term arrhythmia‐free survival [23]. These results warrant cautious interpretation given protocol heterogeneity in ablation strategies across the study period and the inclusion of patients with prior ablation, both of which may dilute treatment effects. Notably, point estimates numerically favored EIVOM in both index and redo subgroups, suggesting possible underpowering rather than absence of benefit.

### 2.2 Impact of EIVOM on Pulmonary Vein Isolation (PVI) and Other Aspects

Beyond the MI, EIVOM may also facilitate PVI and selected adjunctive lesions to a variable extent. Ethanol infusion can acutely modify the LPV region, particularly the lower and anterior aspects of the LPVs, and has been associated with higher first‐pass LPV isolation and lower acute recovery when combined with RFCA [21, 22]. Small mechanistic studies further suggest that EIVOM may reduce complex fractionated atrial electrogram areas and facilitate posterior‐wall isolation in selected settings [28, 29]. However, these substrate‐modifying effects have not consistently translated into improved long‐term rhythm outcomes, indicating that the principal clinical value of EIVOM remains most closely linked to its ability to facilitate durable MI block.

## 3 Efficacy of EIVOM in PeAF Treatment

Although a standardized ablation strategy for PeAF has not yet been established, EIVOM has emerged as a promising therapeutic complement to RFCA. Evidence indicates that adding extra ablation, including linear lesions, does not consistently improve outcomes beyond PVI alone [2, 30]. However, when a durable MI block is achieved, MI‐directed ablation may regain clinical relevance. In 2009, Valderrábano et al. demonstrated that selective VOM ethanol infusion induced immediate LA posterior myocardial chemical injury, achieving local denervation with reduced radiofrequency delivery, suggesting its potential adjunctive value [31]. Generally, EIVOM combined with RFCA improves rhythm control and reduces the recurrence risk in patients with PeAF undergoing an index ablation compared with single RFCA strategies (Table 2). The VENUS trial first demonstrated that EIVOM plus RFCA (PVI + linear ablation) improves long‐term sinus rhythm maintenance over RFCA alone (49.2% vs. 38.0%) [32]. Furthermore, the PROMPT‐AF supported that EIVOM plus RFCA also outperforms standalone PVI, showing a superior 12‐month arrhythmia‐free survival rate (70.7% vs. 61.5%) [33]. Consistent findings were also reported in the Marshall‐PLAN study, with a higher arrhythmia‐free survival rate in patients with a Marshall‐Plan strategy than with PVI alone (86.4% vs. 66.1%) [35]. Although AF burden results have been less consistent across trials, likely reflecting differences in endpoints, monitoring, and lesion‐set execution, these studies collectively suggest that the clinical benefit of EIVOM is closely linked to improved durability of MI block [32, 33, 35, 36].

**Table 2:** A summary of EIVOM's efficacy in PeAF treatment.

Author	RFCA strategy	Follow‐up	Freedom from arrhythmia at 12 months^a^ (E vs. R)	Result
Valderrábano M [32]	PVI, MI, PW, CFAE	At least 30 days of data from implanted monitoring devices^b^ at 6 and 12 months	49.2% (91/185) versus 38.0% (60/158), OR = 0.63, 95% CI 0.41–0.97, *p* = 0.04	EIVOM combined with RFCA increased the likelihood of remaining sinus rhythm at 6 and 12 months.
Lai Y [22]	PVI, MI, RFL, CTI, and CS ablation	24 h Holter recording, at 1, 2, 3, 6, and 12 months, and symptom‐triggered ECGs	84.9% (58/66) versus 67.8% (81/125), *p* < 0.001	Upgraded “2C3L” strategy was associated with higher effectiveness for ablation of PeAF.
Sang C [33]	PVI, MI, RFL, CTI, and CS ablation	A single‐lead ECG wearing for at least 24 h each week for 12 months, also with Holter monitoring, other licensed wearable devices, and symptom‐triggered ECGs	70.7% (174/246) versus 61.5% (153/249), HR = 0.73, 95% CI 0.54–0.99, *p* = 0.045	Upgraded “2C3L” strategy improved freedom from atrial arrhythmias within 12 months compared with PVI alone.
Derval N [34]	PVI, MI, RFL, CTI, and CS ablation when needed	12‐lead electrocardiogram, 24 h Holter recording, and transthoracic echocardiogram at 3, 6, and 12 months	72% (54/75)	Marshall‐PLAN strategy improved sinus rhythm maintenance at 12 months after a single ablation procedure.
Derval N [35]	PVI, MI, RFL, CTI, and CS ablation, floor line when needed	12‐lead electrocardiogram at 3, 6, and 12 months, transthoracic echocardiography at 1 day, 3 and 12 months, and 30 s electrocardiogram recording transmitted every week	86.4% (51/59) versus 66.1 (39/59), *p* = 0.012	Marshall‐PLAN strategy was significantly superior to single PVI strategy at 12 months.
Zuo S [16]	PVI, MI, RFL, CTI, and CS ablation	A 12‐lead ECG, and 24 h Holter recording at 3, 6, and 12 months	82.2% (37/45) versus 68.7% (30/44); *p* < 0.01	EIVOM can reduce acute MI reconnection while improving long‐term rhythm control of PeAF.

*Note:* Data are presented as *n*%, *n*/*N*, absolute value, and mean ± SD.

Abbreviations: CFAE, complex fractionated atrial electrogram; CS, coronary sinus; CTI, cavotricuspid isthmus; E, EIVOM group plus RFCA; ECG, electrocardiogram; EIVOM, ethanol infusion into the vein of Marshall; m, month; MI, mitral isthmus; OS, observational study; PeAF, persistent atrial fibrillation; PVI, pulmonary vein isolation; PW, posterior wall; R, only RFCA group; RCT, randomized controlled trial; RFCA, radiofrequency catheter ablation; RFL, roofline.

^a^ Freedom from any arrhythmias (lasting longer than 30 s) without anti‐arrhythmia drugs after index ablation, except for a 3‐month blanking period

^b^ Implanted monitoring devices include pacemakers, defibrillators, or implanted loop recorders.

Across published meta‐analyses, adjunctive EIVOM has consistently been associated with lower AF/AT recurrence and higher MIBB rates than RFCA alone (Table 3) [37, 38, 39, 40, 41, 42]. These analyses support the clinical value of EIVOM, particularly its effect on improving MIBB. However, the magnitude of rhythm benefit should be interpreted in light of several sources of heterogeneity. First, the evidence base has included both randomized and observational studies, which may introduce potential selection bias and residual confounding. Second, the ablation protocols were not uniform. Beyond PVI and MI ablation, EIVOM was variably combined with roofline, posterior‐wall isolation, CS ablation, or broader substrate modification, making it difficult to isolate its independent contribution to long‐term rhythm outcomes [40]. Furthermore, recurrence assessment differed across studies, including variation in recurrence definitions, follow‐up duration, and rhythm‐monitoring intensity [41, 42]. Intermittent electrocardiogram or 24‐hour Holter monitoring may underestimate recurrence compared with continuous, wearable, or device‐based monitoring, and freedom from recurrence is not directly equivalent to AF burden. Therefore, EIVOM‐associated improvement in MIBB remains consistent across meta‐analyses, whereas the precise extent of long‐term rhythm benefit should be interpreted in relation to procedural strategy, patient characteristics, and surveillance intensity.

**Table 3:** Summary of published meta‐analyses evaluating adjunctive EIVOM in AF treatment.

Author	Year	Study	Patient inclusion	Follow‐up	Outcome	Main finding
Mhanna M [37]	2022	1 RCT 3 OS	PeAF, PaAF, Post‐AFAT; 804 totally	Mostly 3–12 months	EIVOM group was associated with lower AF/AT recurrence (RR = 0.63) and higher MIBB (RR = 1.39), with comparable complication rates.	Adjunctive EIVOM strategy is more effective than conventional catheter ablation with similar safety profiles.
He Z [38]	2021	1 RCT 9 OS	PeAF, PaAF; 1322 totally	Mostly 6–12 months	EIVOM was associated with higher MIBB (RR = 0.58), while lower AF/AT recurrence (RR = 1.50) was observed only in PeAF patients.	Adjuvant EIVOM reduces AF/AT recurrence rate in PeAF patients and increases the success rate of bidirectional MI block.
Li F [39]	2022	1 RCT 5 OS	PeAF, PaAF; 1337 totally	At least 12 months	EIVOM was associated with a higher rate of long‐term rhythm control (RR = 1.28) and successful MIBB (RR = 1.52) with no significant difference in complications.	EIVOM has superior efficacy and comparable safety over ablation alone in AF patients with long‐term follow‐up.
Pranata R [40]	2024	2 RCT 9 OS	PMAT, PeAF, PaAF; 2821 totally	Mostly 12 months	EIVOM was associated with reduced AT recurrence (OR = 0.52) and higher successful MIBB (OR = 3.87).	The addition of EIVOM to ablation increased MIBB and reduced AT recurrence.
Ge WL [41]	2024	1RCT 8 OS	PMAT, PMF, PeAF, PaAF; 2508 totally	Mostly 12 months	EIVOM group showed lower AF/AT recurrence (RR = 0.70) and higher acute MIBB (RR = 1.29).	Adjunctive EIVOM strategies are more effective than conventional catheter ablation.
Itaya ED [42]	2025	4 RCT 16 OS	PMAT, PMF, PeAF, PaAF; 4732 totally	Mostly 12 months	EIVOM was associated with a lower AT recurrence (OR = 0.51) and significantly higher rates of MIBB (OR = 4.41).	EIVOM combined with RFCA was associated with reduced AT recurrence and higher rates of MI block compared to RFCA alone.

Abbreviations: AF, atrial fibrillation; AT, atrial tachycardia; EIVOM, ethanol infusion into the vein of Marshall; MIBB, mitral isthmus bidirectional block; OS, observational study; PaAF, paroxysmal atrial fibrillation; PeAF, persistent atrial fibrillation; PMAT, peri‐mitral atrial tachycardia; PMF, peri‐mitral flutter; RCT, randomized controlled trial.

Observational and single‐center studies have provided supportive real‐world data on adjunctive EIVOM strategies, with reported benefits in 12‐month sinus rhythm maintenance or arrhythmia‐free survival rates broadly consistent with randomized trial findings [43, 44, 45]. Subgroup analyses indicate that the benefit of EIVOM may be more pronounced in patients under 65 years, whereas outcomes appear comparable between patients with and without heart failure, including across different heart failure subtypes [46, 47]. A propensity‐matched study further suggested superior 12‐month sinus rhythm maintenance with an EIVOM‐based strategy compared with PVI alone or PVI plus posterior‐wall isolation [48]. Overall, these real‐world data support the clinical feasibility of EIVOM, but should be interpreted as complementary to randomized evidence.

Furthermore, emerging data indicate that adjunctive EIVOM may be effective in MI‐dependent atrial tachycardias (AT), including perimitral flutter (PMF) and VOM‐related AT. Takigawa et al. reported a higher MIBB rate and more effective PMF termination with EIVOM plus RFCA versus RFCA alone [49]. Lam et al. similarly observed higher acute MI block rate among patients with AF, PMF, and VOM‐related AT [50]. Sang et al. further showed benefit in recurrent perimitral AT, with EIVOM yielding lower recurrence compared with repeat RFCA, particularly in VOM‐related cases [15]. Hence, these findings support EIVOM as an anatomically targeted option for patients refractory to RFCA in the MI‐dependent substrate.

Although the EIVOM‐augmented strategy improves rhythm outcomes, a subset of patients still experiences recurrence. Current evidence does not consistently support a simple phenotype based on conventional clinical variables. Luo et al. reported that EIVOM plus RFCA was associated with superior long‐term rhythm outcome and MI block rate than RFCA alone in PeAF patients with severe LA enlargement [51]. In PROMPT‐AF, the treatment effect was generally consistent across prespecified subgroups, including long‐standing PeAF, LA diameter ≥ 45 mm, larger LA volume, heart failure, reduced left ventricular ejection fraction, and LA low‐voltage areas [33]. Conversely, the VENUS secondary analysis suggested the benefit of EIVOM was more evident when bidirectional perimitral block was achieved and when procedures were performed in high‐volume centers [36]. In the recurrent AF setting, the MARS‐AF study showed only modest benefit of this adjunctive strategy compared with repeat RFCA alone, with no significant difference in freedom from ATs between groups [52]. However, Gunn et al. further observed that recurrence despite durable PVI appeared to be associated with a higher likelihood of freedom from AF at 12 months, suggesting that the incremental value of EIVOM may depend on the dominant mechanism of recurrence and may be limited when recurrence is mainly driven by non‐VOM‐related mechanisms [53]. Unfavorable anatomical factors, including VOM non‐visualization, difficult cannulation, or unfavorable morphology, may also restrict ethanol delivery and reduce its ability to facilitate durable MIBB [35]. Moreover, because EIVOM usually requires complementary RFCA to eliminate residual MA‐side and CS/GCV‐related conduction, inadequate supplemental ablation may also attenuate its clinical benefit. Thus, limited response to EIVOM should be viewed as a combined phenotype involving atrial substrate, recurrence mechanism, VOM anatomy, operator experience, and achievement of electrophysiological endpoints.

### 3.1 Timing Dilemma of EIVOM in AF Treatment

Notably, the optimal timing of EIVOM remains another procedural contention. Rather than representing a minor technical preference, timing may directly affect VOM accessibility, ethanol delivery, and the likelihood of successful MI block. When EIVOM is planned as part of an ablation strategy, current evidence supports a standardized EIVOM‐first workflow, particularly before intra‐CS or MI RFCA. Prior RFCA applications within the CS or adjacent MI region may induce local edema, stenosis, or distortion of the VOM ostium, thereby compromising subsequent VOM identification, cannulation, balloon occlusion, and ethanol infusion. Indeed, Kitamura et al. reported that VOM stenosis occurred in 25% of patients following CS ablation [54]. Clinical studies have further shown that prior EIVOM may increase MI block rates, reduce peri‐mitral AT recurrence, and decrease the need for endocardial or intra‐CS complementary applications [55, 56, 57]. Conversely, some investigators advocate performing EIVOM after general RFCA, arguing that although core EIVOM lesions are highly durable, peripheral borders may evolve over time [58]. Marchese et al. observed that the endocardial lesion extent of EIVOM decreased at 1‐month reassessment compared with immediate post‐infusion measurement, whereas epicardial injury appeared relatively stable [59]. Meanwhile, as EIVOM increases fluoroscopy time, a selective post‐RFCA EIVOM approach may still be suggested to mitigate radiation exposure. However, given the risk of RFCA‐induced VOM stenosis and the accumulating evidence supporting improved procedural efficiency, EIVOM‐first should be considered the preferred default workflow when VOM ethanol infusion is intended.

## 4 Technical Limitations and Safety Considerations of EIVOM

### 4.1 Technical Limitations

As EIVOM demonstrated clinical potential in AF management, the procedural success rate varies from 83.7% to 92.1% [16, 33, 34]. Critically, the morphological variation of VOM was regarded as the primary reason leading to the procedure failure. Except for congenital absence, a high degree of variation existed in the position and shape of the VOM. Failures are mainly attributed to VOM non‐identification, non‐cannulation, or misinjection [60]. On the other hand, the VOM morphology also influences the procedure outcome. Kamakura et al. categorized VOM anatomy into three types, including multi‐branch, large trunk, and non‐branch, and reported that the non‐branch shape was found to be associated with a marked reduction in endocardial scarring (4.7 ± 2.3 cm^2^ with vs. 11.1 ± 5.1 cm^2^ without, *p* < 0.0001), suggesting limited lesion extension [60]. Andronache et al. further highlighted that angiographic geometry also affects procedural success, including the distance from the VOM ostium to the CS and the take‐off angle [25]. As VOM mostly converged 20–50 mm from the opening of the CS, with an average of 140°, the short distance and acute take‐off angle were strongly associated with difficult VOM cannulation.

In addition to anatomic variability, a learning‐curve effect has been observed. The VENUS post‐hoc analysis indicated a volume‐dependent learning curve effect, as long‐term sinus rhythm maintenance rate significantly correlated with institutional experience (high‐volume vs. low‐volume center, *p* = 0.04) [36]. Similar improvements in procedural efficiency and outcomes with increasing case volume were also documented in single‐center series, reinforcing the existence of a learning‐curve dependency for EIVOM implementation [61].

The standardization of ethanol injection also remains an important technical issue (Supporting Information S1: Table 1). Due to the variation in VOM morphology, including diameter, length, tributaries, and leakage risk, the standardization and individualization of injections may be contentious regarding alcohol application. To date, a total of 6–12 mL of ethanol (95% v/v) with slow delivery in divided injections in the distal VOM remains a commonly employed approach as described by Sang et al. in their series study [33, 62]. However, emerging evidence suggests that alternative administration strategies may yield equivalent clinical efficacy. For example, Kong et al. reported that a total ethanol dose > 5.75 mL was related to successful MI block [26]. Therefore, although 6–12 mL currently represents the practical consensus range in clinical studies, the optimal dose should be individualized according to VOM anatomy, including vessel size, length, branching pattern, collateral drainage, and leakage risk. Nevertheless, the endpoint of the injection also fails to develop a shared understanding. Currently, successful EIVOM is usually evaluated by a combination of venographic confirmation of selective ethanol delivery and electroanatomic evidence of ethanol‐induced low‐voltage areas [35, 62]. In this context, intracardiac echocardiography (ICE) may serve as a useful adjunct for real‐time evaluation of ethanol distribution and tissue response. Increased local myocardial echogenicity along the LA ridge and consistent echogenic streaming in the left atrium during VOM ethanol infusion were associated with a larger ethanol‐induced low‐voltage area and a shorter radiofrequency ablation time required to achieve MI block [63, 64]. Thus, ICE may provide complementary information beyond venography, although its role in evaluating successful EIVOM requires further validation.

### 4.2 Safety Profile and Complication Prevention

Although current clinical studies suggest that adjunctive EIVOM does not markedly increase major adverse events compared with RFCA alone, the procedure‐specific complications should be considered given its intravascular ethanol‐delivery nature. Reported procedure‐related events include CS or VOM dissection, VOM perforation, contrast or ethanol leakage, myocardial staining, pericardial effusion or pericarditis, and vascular access complications (Supporting Information S1: Table 2) [60, 65]. In particular, venous injury may occur during guidewire manipulation, balloon advancement, or forceful injection, and has been implicated in the occurrence of pericardial effusion [45]. Meanwhile, incomplete balloon occlusion may permit unintended ethanol leakage or reflux into the CS and, potentially, systemic venous circulation. Most angiographic staining appears to represent localized tissue penetration and may accompany effective lesion formation [66]. However, due to the variable drainage range of VOM, the ethanol‐induced lesions may not be completely spatially predictable. For example, Lu et al. reported incidental left atrial appendage electrical isolation after only 2 mL of alcohol injection [67]. Therefore, post‐infusion voltage mapping should be carefully performed to identify unexpected collateral lesion extension, whereas abnormal extravasation should prompt immediate termination of injection and reassessment. From a procedural standpoint, risk reduction may depend on meticulous CS venography, selective VOM identification, gentle wire/balloon manipulation, stable balloon occlusion, slow low‐pressure ethanol injection, and peri‐procedural surveillance for hemodynamic instability or pericardial effusion. ICE may further facilitate intraprocedural detection of pericardial effusion and guide timely reassessment when complications are suspected [63]. Thus, EIVOM should be regarded as a generally feasible adjunctive strategy, but its safety depends strongly on anatomical recognition, injection technique, and peri‐procedural surveillance.

### 4.3 Potential Impact on LA Function

Beyond procedural and anatomic considerations, potential concerns have been raised regarding the functional consequences of ethanol‐induced scarring on LA performance. Although the relationship between RFCA‐related fibrosis and LA mechanical impairment has been well documented, the corresponding evidence for EIVOM remains limited [68, 69]. Aranyó et al. evaluated EIVOM‐induced structural and functional alterations using late gadolinium–enhanced cardiac magnetic resonance and found no significant deterioration in LA strain, ejection fraction, or emptying fraction at 3 months, aside from a modest reduction in LA volume among 21 patients, supporting the preservation of global atrial function [70]. Likewise, Derval et al. observed a sustained increase in A‐wave velocity during 3‐ to 12‐month follow‐up, suggesting improved atrial contractility after successful EIVOM [35]. Collectively, these findings indicate that, despite its transmural and potentially extensive lesions, EIVOM does not adversely affect, and may even preserve or mildly improve, LA mechanical function within the context of long‐term rhythm control, though larger prospective investigations are warranted to confirm these findings.

## 5 The Role of EIVOM in the Era of PFA

With the increasing use of PFA, the role of EIVOM requires reconsideration. Although PFA provides a nonthermal and relatively myocardial‐selective energy source improving procedural efficiency and has shown feasibility for MI ablation, achieving durable MIBB with PFA alone may remain challenging [71, 72]. La Fazia et al. reported that acute MI block with PFA was achieved in all patients, whereas durable MI block was present in only 5.5% of patients at follow‐up remapping [73]. Moreover, available evidence also suggests PFA applications near the MI may be associated with coronary vasospasm [74]. In this context, EIVOM may retain a complementary role by facilitating durable MIBB and targeting VOM‐related epicardial connections. A small retrospective comparison further suggested that EIVOM plus RFCA may reduce the RFCA burden required for MI block in selected PFA‐based workflows [75]. Nevertheless, further prospective studies directly comparing PFA‐based strategies with EIVOM‐based or hybrid approaches are needed.

## 6 Conclusion

Adjunctive EIVOM has emerged as a promising complement to conventional RFCA, particularly for achieving durable MI block. Current evidence indicates that integrating EIVOM with RFCA significantly enhances long‐term rhythm outcomes, primarily through facilitating durable MI block and reducing residual epicardial conduction. While accumulating evidence supports its therapeutic value, further large multicenter trials with standardized protocols and extended follow‐up are warranted to confirm its durability, optimize procedural integration, and clarify its broader role in comprehensive AF ablation strategies.

## Conflicts of Interest

The authors declare no conflicts of interest.

## Supporting information


**Table S1:** A summary of EIVOM methods.
**Table S2:** Representative studies reporting EIVOM‐related complications.

## Data Availability

Data sharing is not applicable to this article as no data sets were generated or analyzed during the current study.
