# Supplementary material for: Ethanol Infusion Into the Vein of Marshall for Atrial Fibrillation: Clinical Efficacy and Technical Limitations
Source: Clin Cardiol. 2026 Jun 29;49(7):e70398. doi: 10.1002/clc.70398 (PMC13312799; doi:10.1002/clc.70398)
Supplement: Supplementary file 1 — Table S1: A summary of EIVOM methods. Table S2: Representative studies reporting EIVOM‐related complications. [file CLC-49-e70398-s001.pdf]

**Supplement Table 1. A summary of EIVOM methods**

| Author | EIVOM procedural details | | | | |
|---|---|---|---|---|---|
| | Successful rate (%, n/N) | Ethanol concentration (v/v) | Method | Endpoint | Timing |
| Kamakura T [1] | 88.9% (634/713) | 96% | 1–3 mL of ethanol with 3 repeat injections over 1 min | - | Before RFCA |
| Zuo S [2] | 91.1% (41/45) | 95% | Total 6-12 mL of ethanol slowly injected in the distal VOM | - | Before RFCA |
| Valderrábano M [3] | 83.7% (155/185) | 98% | 1 mL of ethanol with up to 4 repeat injections over 2 min, depending on the length of VOM, from distal to proximal | - | Before RFCA |
| Derval N [4] | 92% (75/69) | 98% | Less than 3 mL of ethanol with 3 repeat injections over 1 min, while totally 6-10 mL | Progressive appearance of tissue contrast staining showed in VOM venogram | Before RFCA |
| Derval N [5] | 97% (57/59) | 96% | Total 10 mL of ethanol with 3 separate 1-minute injections | - | Before RFCA |
| Sang C [6] | 85% (209/246) | 95% | Total 6-12 mL of ethanol slowly injected in the distal VOM | - | Before RFCA |
| Lam A [7] | 86% (19/22) | 96% | Totally less than 9 mL of ethanol injected in the distal VOM over 1-2 min | Ongoing PMF showed cycle length prolongation or arrhythmia termination or conduction delay in decapolar CS catheter during LAA pacing | Before RFCA |

| | | | | | |
|---|---|---|---|---|---|
| Kitamura T [8] | 92.6% (50/54) | 96% | 0.5-3 mL of ethanol slowly injected over 1 minute with 1-3 mL of ethanol repeat injected in the same part or another branch, totally less than 12 mL | AT termination or conversion into another AT | Before RFCA |

Data are presented as n%, n/N, absolute value. Abbreviation: EIVOM, ethanol infusion into the vein of Marshall; VOM, vein of Marshall; RFCA, radiofrequency catheter ablation; PMF, peri-mitral flutter; CS, coronary sinus; LAA, left atrial appendage; AT, atrial tachycardias.

**Supplement Table 2. Representative studies reporting EIVOM-related complications**

| Author | EIVOM case[a], n | Pericardial complications[b], n | VOM perforation, n | VOM dissection, n | Contrast leakage, n |
|---|---|---|---|---|---|
| Kamakura T [1] | 713 | 20 | 20 | 68 | 19 |
| Valderrábano M [3] | 185 | 15 | - | - | - |
| Derval N [4] | 75 | 4 | - | 11 | - |
| Sang C [6] | 174 | 9 | - | - | - |
| Luo T [9] | 360 | 7 | - | - | - |
| Leyton-Mange JS [10] | 129 | 6 | 1 | 1 | - |
| Nakashima T [11] | 152 | 0 | - | 1 | - |
| Huang L [12] | 135 | 0 | - | 2 | - |
| Nesti M [13] | 31 | 4 | 3 | 2 | 3 |

Data are presented as absolute value. Abbreviations: EIVOM, ethanol infusion into the vein of Marshall; VOM, vein of Marshall

a: EIVOM case is defined as all patients who received EIVOM, whether successful or not.

b: Pericardial complications encompass pericardial effusion, pericarditis and cardiac tamponade during or following procedure, whether medical intervention is required or not.

c: "-" indicates that the event was not reported or was not separately included in the complication statistics.


1. Kamakura T, Derval N, Duchateau J, et al. Vein of Marshall Ethanol Infusion: Feasibility, Pitfalls, and Complications in Over 700 Patients. *Circ Arrhythm Electrophysiol*. Aug 2021;14(8):e010001. doi:10.1161/circep.121.010001

2. Zuo S, Sang C, Long D, et al. Efficiency and Durability of EIVOM on Acute Reconnection After Mitral Isthmus Bidirectional Block. *JACC Clin Electrophysiol*. Apr 2024;10(4):685-694. doi:10.1016/j.jacep.2023.11.027

3. Valderrábano M, Peterson LE, Swarup V, et al. Effect of Catheter Ablation With Vein of Marshall Ethanol Infusion vs Catheter Ablation Alone on Persistent Atrial Fibrillation: The VENUS Randomized Clinical Trial. *Jama*. Oct 27 2020;324(16):1620-1628. doi:10.1001/jama.2020.16195
4. Derval N, Duchateau J, Denis A, et al. Marshall bundle elimination, Pulmonary vein isolation, and Line completion for ANatomical ablation of persistent atrial fibrillation (Marshall-PLAN): Prospective, single-center study. *Heart Rhythm*. Apr 2021;18(4):529-537. doi:10.1016/j.hrthm.2020.12.023
5. Derval N, Tixier R, Duchateau J, et al. Marshall-Plan Ablation Strategy Versus Pulmonary Vein Isolation in Persistent AF: A Randomized Controlled Trial. *Circ Arrhythm Electrophysiol*. May 2025;18(5):e013427. doi:10.1161/circep.124.013427
6. Sang C, Liu Q, Lai Y, et al. Pulmonary Vein Isolation With Optimized Linear Ablation vs Pulmonary Vein Isolation Alone for Persistent AF: The PROMPT-AF Randomized Clinical Trial. *Jama*. Nov 18 2024;doi:10.1001/jama.2024.24438
7. Lam A, Küffer T, Hunziker L, et al. Efficacy and safety of ethanol infusion into the vein of Marshall for mitral isthmus ablation. *J Cardiovasc Electrophysiol*. Jun 2021;32(6):1610-1619. doi:10.1111/jce.15064
8. Kitamura T, Vlachos K, Denis A, et al. Ethanol infusion for Marshall bundle epicardial connections in Marshall bundle-related atrial tachycardias following atrial fibrillation ablation: The accessibility and success rate of ethanol infusion by using a femoral approach. *J Cardiovasc Electrophysiol*. Sep 2019;30(9):1443-1451. doi:10.1111/jce.14019
9. Luo T, Chen Y, Xiong X, Cheng G, Deng C, Zhang J. Efficacy and safety of the vein of Marshall ethanol infusion with radiofrequency catheter ablation for the treatment of persistent atrial fibrillation in elderly patients. *Front Cardiovasc Med*. 2023;10:1276317. doi:10.3389/fcvm.2023.1276317
10. Leyton-Mange JS, Tandon K, Sze EY, Carpenter CM, Sesselberg HW. The Maine vein of Marshall ethanol experience: learning curve and safety. *J Interv Card Electrophysiol*. Apr 2023;66(3):661-671. doi:10.1007/s10840-022-01378-8
11. Nakashima T, Pambrun T, Vlachos K, et al. Impact of Vein of Marshall Ethanol Infusion on Mitral Isthmus Block: Efficacy and Durability. *Circ Arrhythm Electrophysiol*. Dec 2020;13(12):e008884. doi:10.1161/circep.120.008884
12. Huang L, Gao M, Lai Y, et al. The adjunctive effect for left pulmonary vein isolation of vein of Marshall ethanol infusion in persistent atrial fibrillation. *Europace*. Feb 16 2023;25(2):441-449. doi:10.1093/europace/euac219
13. Nesti M, Luca F, Panchetti L, et al. Impact of Vein of Marshall Ethanol Infusion Combined with Anatomical Ablation for the Treatment of Persistent Atrial Fibrillation: A Long-Term Follow-Up Based on Implantable Loop Recorders. *J Clin Med*. Nov 3 2023;12(21)doi:10.3390/jcm12216916
